# Cranial and lumbosacral hypertrophic pachymeningitis associated with systemic lupus erythematosus: A case report

**DOI:** 10.1097/MD.0000000000004737

**Published:** 2016-09-30

**Authors:** Fei Han, Ding-Rong Zhong, Hong-Lin Hao, Wei-Ze Kong, Yi-Cheng Zhu, Hong-Zhi Guan, Li-Ying Cui

**Affiliations:** a Department of Neurology; b Department of Pathology, Peking Union Medical College Hospital, Chinese Academy of Medical Sciences, Beijing 100730, China.

**Keywords:** hypertrophic cranial pachymeningitis, hypertrophic spinal pachymeningitis, magnetic resonance imaging, pathology, systemic lupus erythematosus

## Abstract

**Background:**

Hypertrophic pachymeningitis (HP) is a chronic disease characterized by inflammatory hypertrophy and fibrosis of dura mater. It can be divided into cranial and spinal forms depending on the location of the lesion. HP involving 2 separate sites simultaneously is quite uncommon.

**Case summary:**

This study presents a case of a 49-year-old woman with pathologically confirmed cranial and lumbosacral hypertrophic pachymeningitis associated with systemic lupus erythematosus (SLE), which is a rare etiology of HP. She experienced persistent numbness and pain of the left lower limb, followed by headache and seizures. In laboratory tests, levels of erythrocyte sedimentation rate and C-reactive protein were elevated, and antinuclear antibodies and anti–double-strand deoxyribonucleic acid (DNA) antibodies were detected. Magnetic resonance imaging revealed dural thickening with homogenous gadolinium enhancement both at lumbosacral level and over cerebral convexities. Histology suggested chronic inflammation in spinal dura mater with extensive fibrosis, dense lymphoplasmacytic infiltrate, and focal vasculitis. Treatment with corticosteroids and cyclophosphamide was started with significant clinical and radiological improvement.

**Conclusion:**

HP is etiologically heterogeneous. Despite its rarity, SLE should be considered in the differential diagnosis of HP. Early recognition and therapy may provide an optimal outcome.

## 1 Introduction

Hypertrophic pachymeningitis (HP) is a rare disorder characterized by marked inflammatory hypertrophy of the dura mater that provokes neurological symptoms. It may be caused by different diseases including infections, autoimmune diseases, and tumors, or being labeled idiopathic in the absence of an identifiable cause. Most cases involved the intracranial or the spinal dura mater alone. This is the first report, of which we are aware, that presents a case of HP with 2 separate sites involved as initial presentation of systemic lupus erythematosus (SLE).

## 2 Case presentation

**Figure 1:**
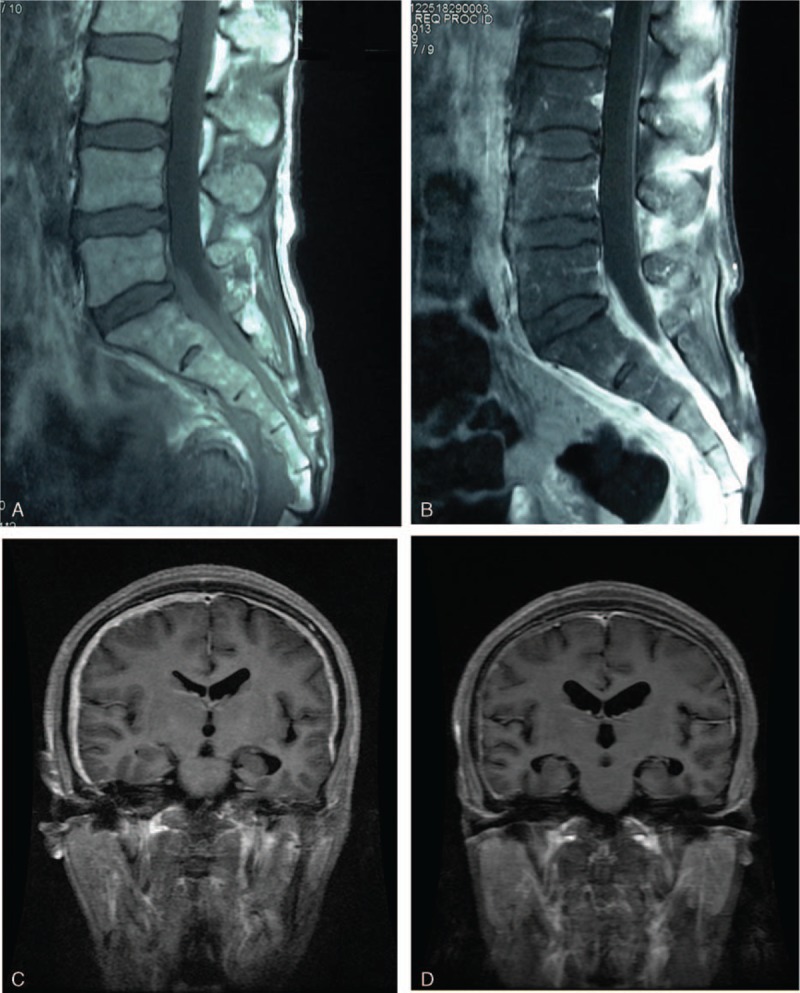
Lumbar magnetic resonance imaging (MRI) showed dural thickening (A) with homogenous gadolinium enhancement (B). Brain MRI revealed diffuse pachymeningeal enhancement over both cerebral convexities (C). Dural thickening greatly resolved 1 year after treatment with steroids and immunosuppressant (D).

A 49-year-old previously healthy woman complained of gradually progressive left buttock swelling together with intermittent low fever for 1 year. She felt numbness and pain radiating to the posterior part of the left leg without obvious weakness. Lumbar magnetic resonance imaging (MRI) in the outside hospital (Fig. 1A and B) showed dural thickening with homogenous gadolinium enhancement and tissue swelling of the left buttock. The patient was treated with antibiotics empirically without symptomatic improvement.

Four months before being admitted to our hospital, she experienced severe headache and diplopia, accompanied with episodes of generalized tonic seizures and intermittent psychiatric symptoms, including hallucinations and disorganized thinking.

On examination, the patient was alert. She presented with bilateral exophthalmos with conjunctival congestion. Slit lamp examination showed peripheral ulcerative keratitis and uveitis, which were indicative of systemic inflammatory disease. Cranial nerve examination revealed incomplete bilateral third and sixth nerve palsies and left peripheral facial palsy. The muscle strength of the distal left lower limb was grade 4/5, while other limbs were normal. Achilles tendon reflexes were reduced bilaterally, and Lasegue sign on the left side was positive. Sensory tests revealed decreased pinprick sensation at L5 to S1 level in the left leg. The rest of the neurological examination and systemic examination were normal.

Brain MRI (Fig. 1C) demonstrated linear dural thickening and diffuse pachymeningeal enhancement over both cerebral convexities, with abnormal signal in the subcortical region of the right frontal lobe, which might have resulted from obstruction of venous reflux caused by the diffuse lesions of dura mater.

Routine blood tests were normal except for mild thrombocytopenia. Erythrocyte sedimentation rate and C-reactive protein were both elevated. Antinuclear antibodies showed 1:640 positive and anti–double-strand deoxyribonucleic acid (DNA) antibodies were detected as well as the presence of hypocomplementemia (C3 0.666 and C4 0.082 g/L; normal range >0.73 and 0.1 g/L). Angiotensin-converting enzyme, antineutrophil cytoplasmic antibodies (ANCA), and rheumatoid factors were negative. Serum immunoglobulin G (IgG)4 level was within normal ranges. Infectious workup including syphilis, HIV, tuberculosis, and brucella were negative as well. Cerebrospinal fluid (CSF) pressure was elevated (>330 mmH_2_O). CSF analysis revealed 82 leukocytes/mm^3^ with lymphocytic predominance, protein of 1.89 g/L, and normal glucose level. Bacterial, fungal, and acid-fast bacilli cultures were all negative. No malignant cells were found on CSF cytology.

**Figure 2:**
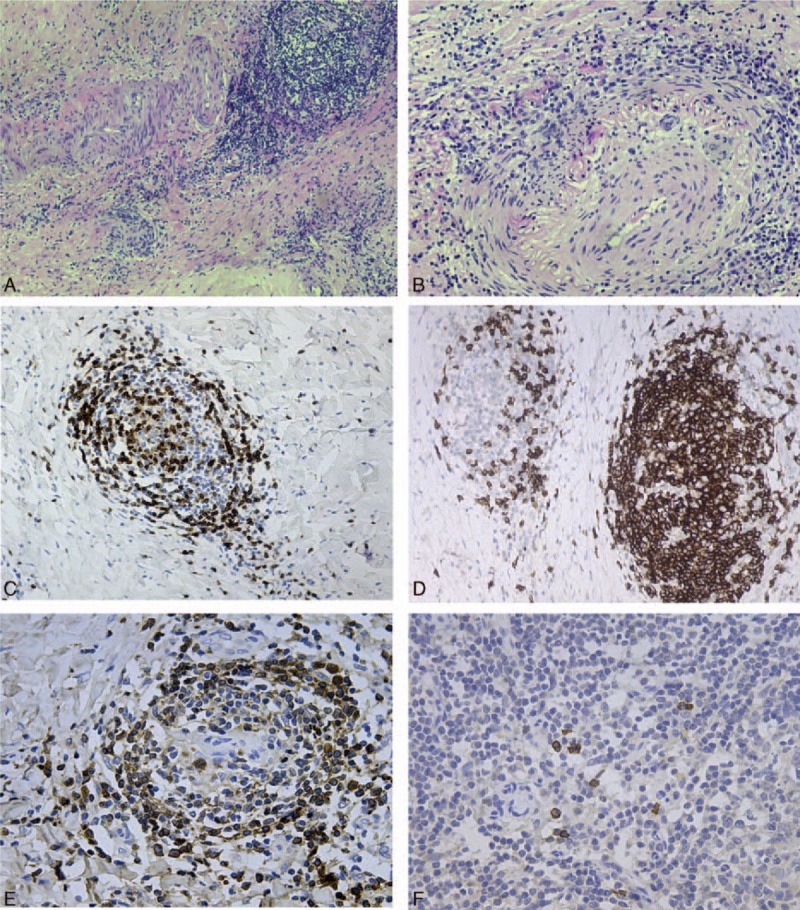
Histological examination revealed chronic inflammation in spinal dura mater with extensive fibrosis, dense lymphocyte, and plasma cell infiltration (A) and focal vasculitis (B). Immunohistochemical analysis demonstrated CD3^+^ T cell (C), CD20^+^ B cell (D), and IgG positive plasma cell (E) infiltration. IgG4 was positive in a fraction of plasma cells (F) with the IgG4-to-IgG ratio 10%. (A ×100, B ×200, C ×200, D ×200, E ×400, and F ×400). IgG = immunoglobulin G.

Sacrococcygeal vertebrae resection was performed for decompression, and surrounding tissue was obtained for pathological diagnosis. There was a large amount of firm tissue adherent to the ventral dural sac, compressing the lumbosacral nerve roots. Histological examination (Fig. 2) revealed chronic inflammation in spinal dura mater with extensive fibrosis, dense lymphoplasmacytic infiltrate, and focal vasculitis. Immunostaining for IgG4 was positive. The IgG4-to-IgG ratio was 10%.

**Figure 3:**
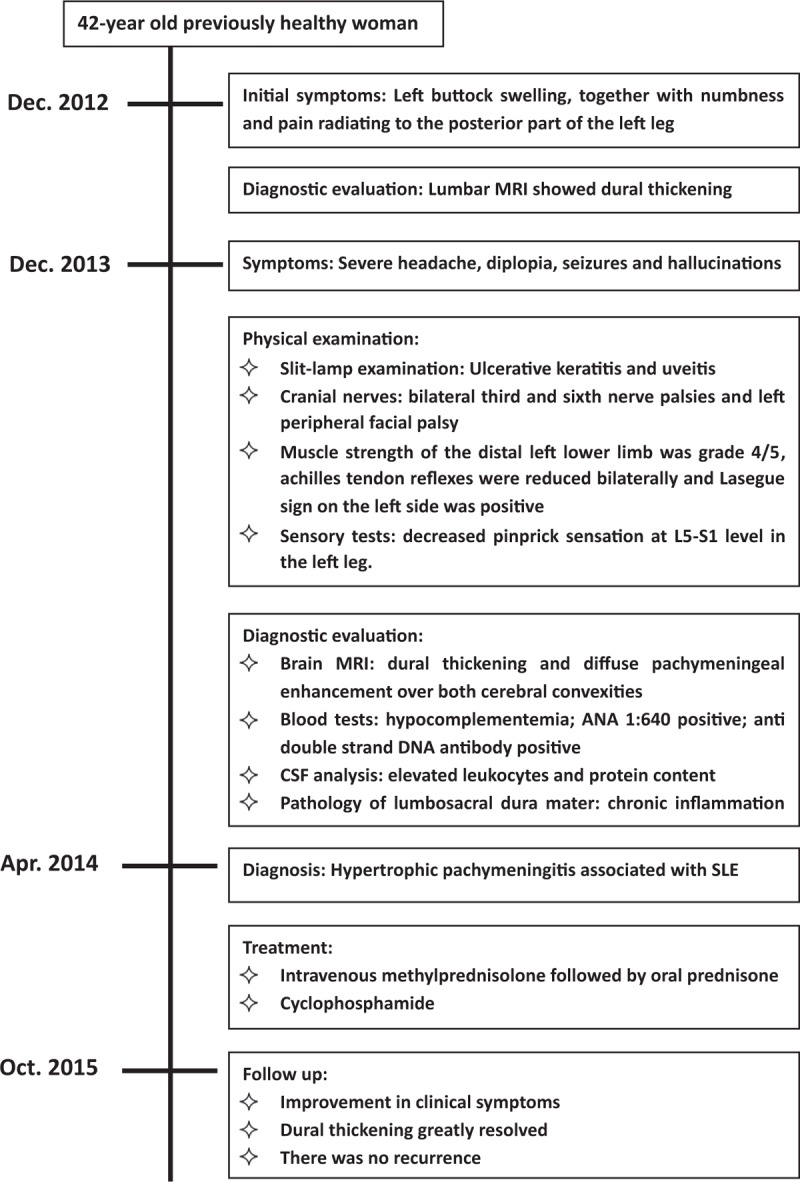
Timeline of diagnosis, interventions, and outcomes.

Based on the clinical, serological, and imaging findings, a diagnosis of hypertrophic pachymeningitis associated with SLE was made (Fig. 3). The patient was treated with intravenous methylprednisolone followed by maintenance treatment with oral prednisone 60 mg daily. Intrathecal injection of dexamethasone was given simultaneously, and immunotherapy was intensified with cyclophosphamide. The patient showed improvement in clinical symptoms, as well as a significant reduction of CSF leucocytes and protein content. Dural thickening was greatly resolved (Fig. 1D). There was no recurrence.

## 3 Discussion

This is a female adult with pathologically confirmed cranial and lumbosacral hypertrophic pachymeningitis associated with SLE. The patient had good response to immunotherapy with complete recovery.

Hypertrophic pachymeningitis is a chronic inflammatory disease, demonstrating local or diffuse thickening of the dura mater. HP can be divided into hypertrophic cranial pachymeningitis (HCP) and hypertrophic spinal pachymeningitis depending on the location of the lesion. It occurs in either of these areas alone or as a craniospinal form. The craniospinal form described in the literature mainly occurs when cranial HP extends caudally to the cervical spine. HP with 2 separated sites involved, like the present case, is extremely rare. In the medical literature, there is only 1 case of idiopathic HP, affecting the lumbosacral spinal dura and the right parietal dura mater at a 5-year interval.^[1]^ Chronic headache, cranial neuropathies, and symptoms due to sinus stenosis or occlusion are the most common symptoms in HCP. The spinal form presents as a chronic progressive disease with manifestations of radiculopathy, myelopathy, or a combination of both. Diagnostic process should rely primarily on gadolinium-enhanced MRI, CSF analysis, and meningeal biopsy.

HP is etiologically heterogeneous in association with a variety of conditions, including infectious diseases such as tuberculosis, syphilis and fungal infection, autoimmune disorders such as ANCA positive vasculitis, IgG4-related disease, rheumatoid arthritis, undifferentiated connective tissue disease, and sarcoidosis. Other factors subsume neoplastic diseases, intrathecal drug administration, trauma, and others. Finally, idiopathic HP can be established as a diagnosis of exclusion with typical pathology. A nationwide survey of HP in Japan demonstrated that idiopathic HP was most common (accounts for 44%), while ANCA-associated HP, especially granulomatosis with polyangiitis, was the most frequent coexisting disease of secondary HP (accounts for 34%), followed by IgG4-related HP (accounts for 8.8%).^[2]^

SLE-related intracranial and spinal hypertrophic pachymeningitis is extremely rare. To the best of our knowledge, only 3 cases have been reported in the medical literature, with one of them resulting from CSF hypovolemia.^[3–5]^ This is the first case of a patient with SLE suffering from HP with 2 separate sites involved. In our case, CSF inflammation was evidenced by elevated CSF leukocytes and protein. Biopsy showed increased lymphocytic infiltration with focal vasculitis, which is consistent with the pathological process of neuropsychiatric SLE. However, it is still not clear how SLE is responsible for the pathogenesis of HP.

Alternative etiologies, including IgG4-related HP, were considered initially in this case. Hallmark histopathological features of IgG4-related HP are lymphoplasmacytic infiltration of IgG4-positive plasma cells (IgG4–IgG ratio greater than 40% or more than 10 cells per high-power field), storiform fibrosis, and obliterative phlebitis.^[6]^ However, staining for IgG4 at immunohistochemical analysis in our case was not consistent with the diagnostic criteria of IgG4-related HP.

Corticosteroids, mostly methylprednisolone pulse therapy followed by oral administration, were administered as the first choice for both HP and SLE. Steroid doses should be carefully tapered in order to avoid relapse. Combinational therapy with immunosuppressants, including cyclophosphamide, mycophenolatemofeil, and azathioprine, could be helpful.

## 4 Conclusion

To summarize, HP is an extremely rare form of nervous system manifestation in SLE patients, especially with 2 separated sites involved. We hope our report will raise awareness of SLE in the differential diagnosis of patients with cranial or spinal dura mater thickening. We recommend that in these cases steroids and immunosuppressants may be considered as first-line treatments, which may provide an optimal outcome.
